# Factors influencing axial elongation in myopic children using overnight orthokeratology

**DOI:** 10.1038/s41598-023-34580-3

**Published:** 2023-05-12

**Authors:** Zhu Huang, Wei Zhao, Ying-zheng Mao, Shan Hu, Chi-Xin Du

**Affiliations:** 1 Department of Ophthalmology, The First Affiliated Hospital, College of Medicine, Zhejiang University, Hangzhou, 310003 China; 2 College of Medicine, Zhejiang University, Hangzhou, 310030 China

**Keywords:** Diseases, Medical research, Risk factors

## Abstract

Several factors influence axial length in children with myopia treated using overnight orthokeratology. To identify these factors, this retrospective study collected axial length and corneal aberration data on 78 eyes before and 1-year after orthokeratology. Patients were divided according to axial elongation (cut-off, 0.25 mm/year). Baseline characteristics included age, sex, spherical equivalent refraction, pupil diameter, axial length, and orthokeratology lens type. Corneal shape effects were compared through tangential difference maps. Group differences in higher-order aberrations of a 4 mm zone were compared at baseline and 1-year following therapy. Binary logistic regression analysis was conducted to identify the variables determined for axial elongation. Significant differences between both groups included the initial age of wearing orthokeratology lenses, type of orthokeratology lens, size of central flattening area, corneal total surface C12 (1-year), corneal total surface C8 (1-year), corneal total surface spherical aberration (SA) (1-year root mean square [RMS] values), change in total corneal surface C12, and change in front and total corneal surface SA (RMS values). The age when wearing an orthokeratology lens was the most important factor influencing axial length in children with orthokeratology-treated myopia, followed by lens type and change in the C12 of the total corneal surface.

## Introduction

The prevalence of myopia among school-aged children has rapidly increased worldwide, especially in East Asia^1–3^. A 2019 report from Wuhan, China, found that the prevalence of myopia increased from 14.92% in 6-year-olds to 86.78% in 12-year-olds^4^. The rapid development of childhood myopia can easily cause high myopia in adulthood, usually accompanied by macular degeneration, retinal detachment, cataracts, glaucoma, and other visual impairment diseases^5–8^. Therefore, controlling myopia progression in school-aged children is important to reduce its severity and complications.

Orthokeratology (OK) is one of the most effective methods for reducing axial elongation and slowing the progression of myopia in children^9–11^. Wearing an OK lens overnight can flatten the central cornea through reverse geometric design, creating a flat central treatment zone surrounded by a steep mid-peripheral ring zone^12^. The flat central treatment zone can improve the patient’s naked vision throughout the day, and the steep mid-peripheral ring zone creates peripheral defocus to control myopia progression^13^.

However, the effects of reducing axial elongation with OK treatment vary from 30 to 52% compared with single-vision spectacles^9,11,14^. The factors potentially affecting the efficacy of OK for retarding childhood myopia progression include the initial age of wearing OK lenses^9,11,14–16^, baseline spherical equivalent refractive error (SER)^11,15–18^, pupil size^16,19^, and high-order aberrations^16,20–23^. Therefore, this study focused on identifying factors that determine the therapeutic effects of OK on slowing childhood myopia progression.

## Methods

### Patients

This retrospective study reviewed the medical records of children with myopia who underwent OK lens treatment at The First Affiliated Hospital of Zhejiang University (Hangzhou, Zhejiang) from January 2021 to December 2022. In total, 78 children (78 right eyes) with myopia met the inclusion criteria and were classified into the effective group (n = 47 eyes) if axial elongation was ≤ 0.25 mm/year; the cut-off point was selected according to Chen et al.^24^, who defined that a fast progressor was a person who had an increase of > 0.25 mm in axial elongation after 1-year of treatment with OK lens; otherwise, they were classified into the ineffective group (n = 31 eyes). This study adhered to the tenets of the Declaration of Helsinki and was approved by the ethics committee of The First Affiliated Hospital, College of Medicine, Zhejiang University. Data were collected from the hospital records, and no patient involvement was required. The ethics committee of The First Affiliated Hospital, College of Medicine, Zhejiang University, waived the requirement for informed consent owing to the retrospective nature of this study.

### Inclusion and exclusion criteria

The inclusion criteria for the study were (1) an age of 8–15 years at baseline, (2) cycloplegic autorefraction (SER) from − 5.00 to − 0.75 dioptre (D) in both eyes, (3) astigmatism (cycloplegic autorefraction) ≤ 1.50 D in both eyes, (4) anisometropia (cycloplegic autorefraction) ≤ 1.50 D, (5) monocular best corrected visual acuity 0.00 logMAR or better, (6) overnight treatment with OK lens, and (7) a follow-up time ≥ 365 days. The exclusion criteria were (1) strabismus and binocular vision abnormalities, (2) ocular and systemic abnormalities, (3) use of medications that might affect refractive development, (4) history of other contact lens use, and (5) incomplete data.

### OK lenses

**Figure 1:**
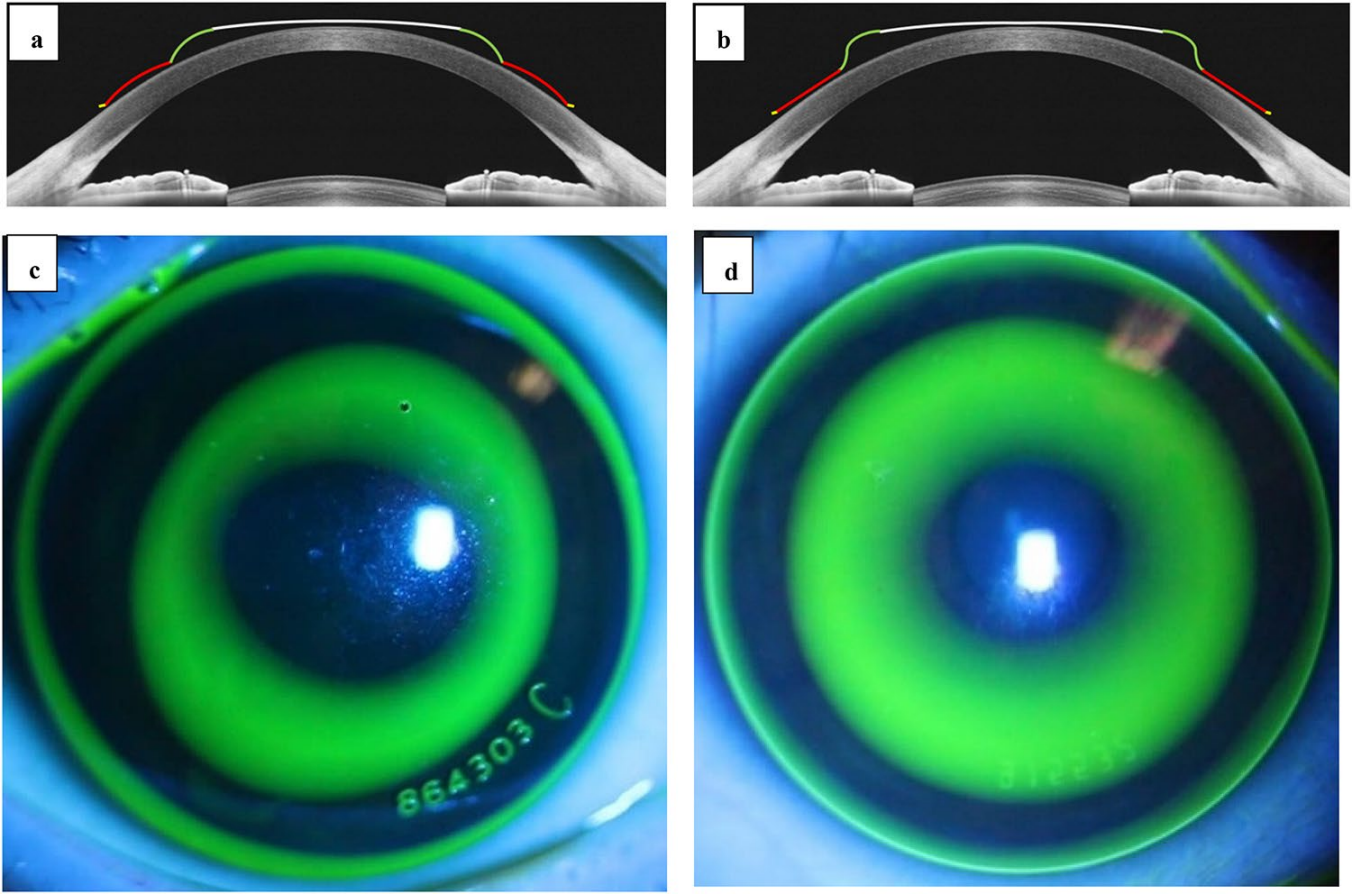
Details of the VST and CRT lens. (**a**) diagram of the VST orthokeratology, which is composed of 4–5 arc segments, mainly including base curve, reverse curve, alignment curve, and peripheral curve. (**b**) diagram of the CRT orthokeratology, which is a three-zone design, including central spherical zone, the S-shaped return zone, and a non-curving landing zone. (**c**) Example VST lens Fluorescein pattern. (**d**) Example CRT lens Fluorescein pattern.

There are two types of traditional OK lens design: vision shaping treatment (VST), as designed by Boston, and corneal refractive therapy (CRT) design, the design patent of Paragon. The parameters of the OK lens designed by VST comprises 4–5 arc segments, mainly including the base curve, reverse curve, alignment curve, and peripheral curve. The CRT lens design comprises three areas, including the central spherical zone, S-shaped return zone, and a non-curving landing zone (More information about the optical design can be found in the supplier's package insert. Paragon Z CRT, Package Insert, available online at: https://www.accessdata.fda.gov/cdrh_docs/pdf5/P050031c.pdf.). The schematic diagram and fluorescein pattern of the VST and CRT designs is presented in Fig. 1.

Two types of OK lenses were used in this study: the Alpha OK lenses (Alpha Corp., Nagoya, Japan), which has a VST design with a nominal degree of transmissibility for Oxygen (Dk) of 104 × 10^−11^ (cm^2^/s) (mL O_2_/mL × mmHg) (ISO/Fatt)^23^, and the Paragon CRT (Paragon Vision Sciences; Mesa, Arizona), which has a CRT design with a nominal Dk of 100 × 10^−11^ (cm^2^/s) (mL O_2_/mL × mmHg) (ISO/Fatt)^25^.

All patients were followed-up according to the standard process of the Ophthalmology Department of the First Affiliated Hospital of Zhejiang University. The OK lenses were fitted following the manufacturer recommendations. The patient was recommended to wear the OK lens for at least 8 consecutive hours every night after the lens was fitted. Patients were usually examined at 1 day, 1 week, and 1 month after wearing the OK, and follow-up was scheduled every 3 or 6 months subsequently. Visual acuity was measured at each visit, and a slit-lamp examination was performed to assess OK lens integrity and eye health. Axial length (AL) was measured every 6 months. Due to the heterogeneity of AL measurement intervals in clinical practice, we analysed only the 1-year values for the AL and number of days of wearing OK lenses. The change in the 1-year AL was calculated by subtracting the 1-year AL from the baseline value, multiplying by 365, and dividing by the number of days for which the OK lens was worn^26^. During the review period, lens wearing stopped for no more than 30 days on end for all patients.

### AL and pupil diameter measurements

AL and pupil diameter were measured using optical ocular biometry (OA-2000, Tomey, Japan). The value of each parameter was calculated as the average of 10 consecutive measurements.

### Refraction measurements

Cycloplegic autorefraction was measured using an auto-kerato-refractometer (ARK-1; Nidek, Japan) 35 min after the instillation of four drops of 0.5% tropicamide. Three successive measurements were performed, and their averages were used as representative values.

### Corneal shape effect measurements

**Figure 2:**
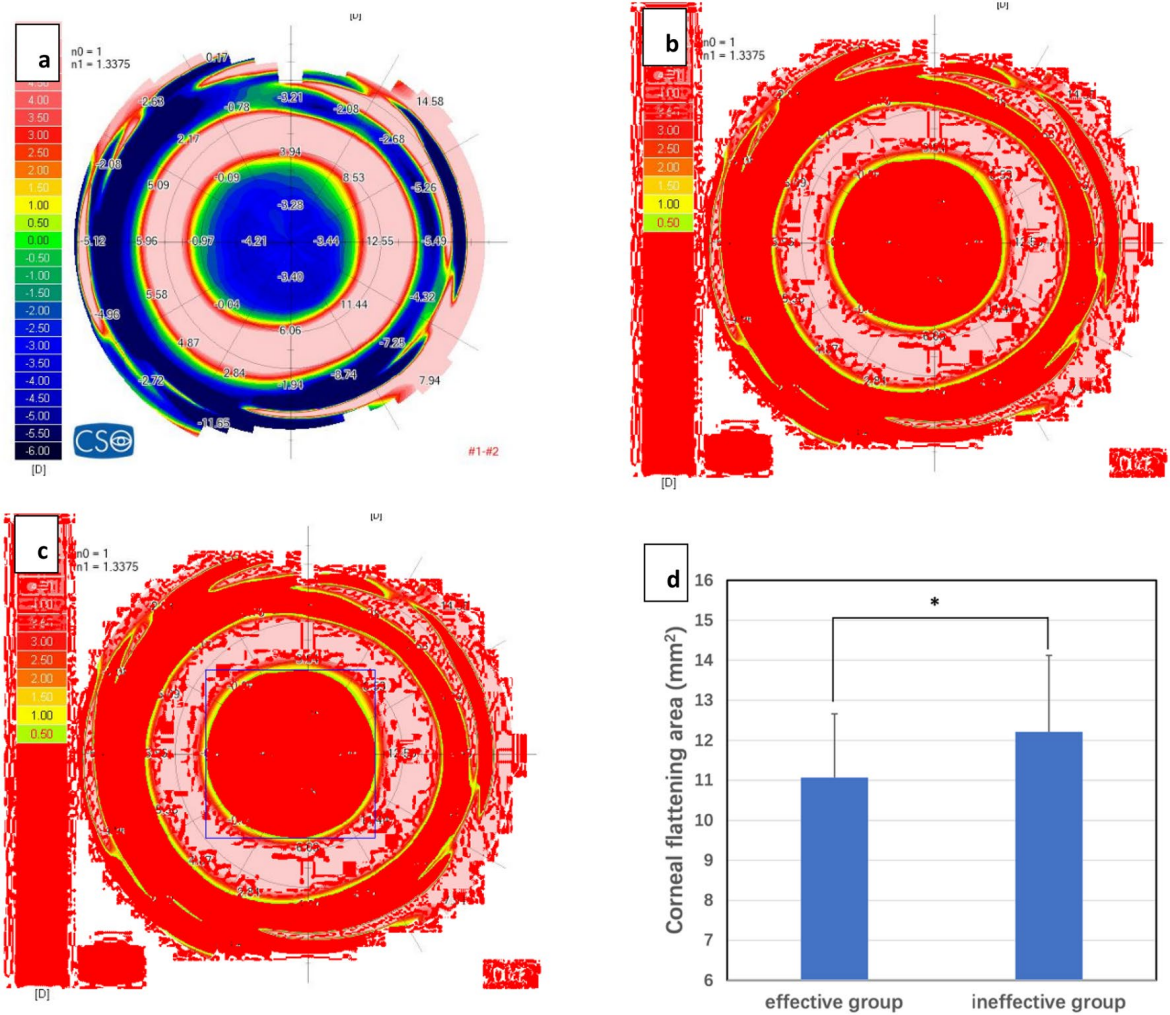
Tangential difference maps were using 3-month topography minus baseline topography. The corneal flattening area (CFA) in the tangential difference maps was located within the central green ring, which means power ≤ 0D (**a**). The color of CFA was converted to red by ImageJ software, and the square value of CFA was also calculated by ImageJ software (**b**). A rectangular border was added outside of CFA (**c**), the eccentricity value was calculated by distance between the center of the rectangle and the center of the coordinate axis. (**d**) the square value of CFA was smaller in the effective group than the ineffective group.

Tangential difference maps were created using 3-month topography values minus baseline topography values. The corneal flattening area (CFA) in the tangential difference maps was located within the central green ring, implying a power of ≤ 0 D (Fig. 2a). The colour of CFA was converted to red using ImageJ software (version 1.48; National Institutes of Health) (Fig. 2b), and the square value of CFA was calculated. A rectangular border was added outside the CFA (Fig. 2c). The eccentricity value was calculated as the distance between the centre of the rectangle and centre of the coordinate axis.

### Higher-order aberration measurements

Higher-order aberrations of the front and total corneal surfaces for a 4 mm zone were measured simultaneously using the Sirius Scheimpflug–Placido topographer (CSO, Italy). Values were collected for C7 (vertical coma), C8 (horizontal coma), and C12 (spherical aberration, SA), the three Zernike polynomials, and the root mean square (RMS) values for coma aberration (coma), SA, and total higher-order aberration were calculated.

### Data collection

Data were collected retrospectively from the clinical records of 78 eyes for statistical analysis, including the initial age at OK lens fitting, sex, baseline SER, baseline AL readings, pupil size, OK lens type, square value and eccentric value of the corneal flattening zone, and high-order aberrations of the front and total corneal surfaces, as detailed below.

### Statistical analysis

Depending on their characteristics, the data were described as frequencies, mean ± standard deviation, or median (minimum–maximum). Statistical analyses were performed to determine differences between all groups. The chi-square or modified chi-square test was used to evaluate categorical variables. Two independent sample t-tests or the Mann–Whitney U test was used to evaluate quantitative variables for analysing significant differences between groups. Spearman or Pearson correlation analysis was used to evaluate the relationship between the two variables. The relationships between risk factors and myopia control were evaluated using a bivariate logistic regression. The covariates in the bivariate logistic regression model included the initial age of OK lens fitting, OK lens type, square value of CFA, and change in SA (RMS value) on the total surface. A *P* value < 0.05 was considered statistically significant. All statistical tests were performed using SPSS Statistics for Windows version 19.0 (SPSS Inc., Chicago, IL, USA).

## Results

### Subjects and their characteristics

The study included 78 eyes that met the inclusion and exclusion criteria between 1 January 2021 and 31 December 2022. The eyes were divided into the effective (47 eyes) and ineffective groups (31 eyes). Table 1 presents the patients’ demographic data. The average initial age at OK lens fitting was 10.33 ± 1.60 years, and 48.7% of patients were male. The average initial SER was 2.45 ± 1.03 D, the initial AL was 24.57 ± 0.53 mm, and the pupil diameter was 3.86 ± 0.52 mm. The two groups had similar values for sex distribution, SER, AL, and pupil diameter (*P* > 0.05). However, there were significant differences between both groups for the initial age at OK lens fitting (*P* < 0.001) and OK lens type (*P* = 0.003).

**Table 1:** Basic Demographics (N = 78).

	Total	Effective Group (n = 47)	Ineffective Group (n = 31)	*P*
Age (years)	10.33 ± 1.60	10.93 ± 1.65	9.42 ± 0.97	< 0.001*
Sex (male)	38 (48.7)	19 (40.4)	19 (61.3)	0.071^†^
Female	40 (51.3)	28 (59.6)	12 (38.7)	
SER(D)	2.45 ± 1.03	2.41 ± 1.13	2.50 ± 0.87	0.688*
AL (mm)	24.57 ± 0.53	24.65 ± 0.51	24.44 ± 0.54	0.080*
PD (mm)	3.86 ± 0.52	3.77 ± 0.55	4.00 ± 0.45	0.060*
Type (CRT)	50 (64.1)	24 (51.1)	26 (83.9)	0.003^†^
Alpha	28 (35.9)	23 (48.9)	5 (16.1)	

Data are presented as n (%) or mean ± standard deviation. *P* < 0.05 was considered statistically significant.

*AL* axial length, *D* diopter, *PD* pupil diameter, *SER* spherical equivalent refraction.

*Two independent samples t-test, ^†^Chi-square test.

### Changes in visual acuity

After wearing an OK lens for 1-year, the visual acuity of the naked eye increased considerably. However, there were no differences between both groups at baseline (*p* = 0.326) and 1-year postoperatively (*p* = 0.507).

### Changes in high-order aberration measurements

After wearing the OK lens for 1-year, higher-order corneal aberration increased considerably. Higher-order aberrations of the front and total corneal surfaces in the 4 mm zone were compared between both groups at baseline and postoperatively (Table 2). The effective group showed more negative values of C12 and C8 of the total corneal surface at 1-year postoperatively than at baseline (*P* = 0.023, C12; *P* = 0.031, C8). The *P* value of the SA (RMS value) was similar to that of C12.

**Table 2:** Higher-order aberrations of front corneal surface and total corneal surface in 4 mm zone at baseline and 1-year later (N = 78).

		Baseline			One year later		
		Effective group (n = 47)	Ineffective group (n = 31)	P*	Effective group (n = 47)	Ineffective group (n = 31)	P*
Front	C7	− 0.01 (− 0.15–0.14)	− 0.04 (− 0.18–0.07)	0.107	− 0.07 (− 0.91–0.58)	− 0.08 (− 0.46–0.15)	0.610
	C8	0.04 (− 0.09–0.11)	0.03 (− 0.04–0.09)	0.353	− 0.11 (− 0.57–0.14)	− 0.08 (− 0.33–0.32)	0.147
	C12	− 0.03 (− 0.09–0.09)	− 0.03 (− 0.08–0.01)	0.284	− 0.13 (− 0.52–0.07)	− 0.10 (− 0.24–0.04)	0.144
	Coma (RMS)	0.06 (0.01–0.16)	0.07 (0.00–0.18)	0.882	0.21 (0.02–1.04)	0.17 (0.04–0.47)	0.277
	SA (RMS)	0.03 (0.00–0.09)	0.03 (0.00–0.08)	0.402	0.13 (0.01–0.52)	0.10 (0.00–0.24)	0.118
	tHOA (RMS)	0.13 (0.07–0.94)	0.13 (0.06–0.84)	0.886	0.32 (0.09–1.18)	0.31 (0.10–0.79)	0.295
Total	C7	− 0.02 (− 0.17–0.11)	− 0.01 (− 0.16–0.06)	0.717	− 0.11 (− 0.92–0.6)	− 0.12 (− 0.47–0.31)	0.894
	C8	0.05 (− 0.07–0.10)	0.05 (− 0.02–0.09)	0.882	− 0.11 (− 0.58–0.15)	− 0.07 (− 0.27–0.19)	0.031
	C12	− 0.03 (− 0.08–0.09)	− 0.03 (− 0.08–0.00)	0.806	− 0.16 (− 0.53–− 0.06)	− 0.14 (− 0.23–− 0.04)	0.023
	Coma (RMS)	0.06 (0.01–0.17)	0.07 (0.00–0.17)	0.661	0.22 (0.01–1.05)	0.17 (0.03–0.47)	0.191
	SA (RMS)	0.03 (0.00–0.09)	0.03 (0.00–0.08)	0.527	0.16 (0.06–0.53)	0.14 (0.04–0.23)	0.023
	tHOA (RMS)	0.12 (0.06–0.30)	0.13 (0.08–0.23)	0.976	0.30 (0.09–1.20)	0.26 (0.10–0.55)	0.136

Data are presented as median (minimum ~ maximum). *P* < 0.05 was considered statistically significant.

*Mann–Whitney U test. C7: Zernike polynomial of vertical coma. C8: Zernike polynomial of horizontal coma. C12: Zernike polynomial of spherical aberration. *Coma*coma aberration, *RMS* root mean square, *SA* spherical aberration, *tHOA* total higher-order aberration.

Changes in higher-order aberrations of the front and total corneal surfaces in the 4 mm zone at baseline and 1-year postoperatively were compared between both groups (Table 3). The effective group showed a more negative change in C12 at the total corneal surface (*P* = 0.025). Moreover, the SA (RMS values) of the effective group showed a significant change at the front and total corneal surface (front *P* = 0.035; total *P* = 0.043).

**Table 3:** Change of higher-order aberrations of front corneal surface and total corneal surface in 4 mm zone between 1-year later and baseline (N = 78).

		Effective group (n = 47)	Ineffective group (n = 31)	P*
Front	C7	− 0.05 (− 0.84 to 0.62)	− 0.05 (− 0.38 to 0.23)	0.779
	C8	− 0.15 (− 0.68 to 0.08)	− 0.13 (− 0.33 to 0.27)	0.253
	C12	− 0.10 (− 0.52 to 0.07)	− 0.05 (− 0.23 to 0.06)	0.051
	Coma (RMS)	0.13 (− 0.09 to 0.95)	0.13 (− 0.03 to 0.36)	0.272
	SA (RMS)	0.09 (− 0.02 to 0.51)	0.05 (0.00 to 0.23)	0.035
	tHOA (RMS)	0.16 (− 0.48 to 1.05)	0.09 (− 0.27 to 0.39)	0.100
Total	C7	− 0.10 (− 0.83 to 0.63)	− 0.09 (− 0.37 to 0.27)	0.858
	C8	− 0.16 (− 0.68 to 0.09)	− 0.12 (− 0.30 to 0.19)	0.088
	C12	− 0.14 (− 0.54 to − 0.01)	− 0.11 (− 0.22 to − 0.01)	0.025
	Coma (RMS)	0.15 (− 0.04 to 0.95)	0.10 (− 0.04 to 0.36)	0.168
	SA (RMS)	0.13 (0.01 to 0.52)	0.11 (0.01 to 0.22)	0.043
	tHOA (RMS)	0.15 (0.00 to 1.05)	0.11 (− 0.01 to 0.39)	0.173

Data are presented as median (minimum ~ maximum). *P* < 0.05 was considered statistically significant.

*Mann–Whitney U test. C7: change of Zernike polynomial of vertical coma. C8: change of Zernike polynomial of horizontal coma. C12: change of Zernike polynomial of spherical aberration. *Coma* change of coma aberration, *RMS* root mean square, *SA* change of spherical aberration, *tHOA* change of total higher-order aberration.

### Corneal shape effect differences

After wearing the OK lens for 3 months, the square value of CFA was significantly smaller in the effective group than in the ineffective group (11.07 ± 1.59 and 12.21 ± 1.91 mm^2^, respectively; *P* = 0.006; Fig. 2d). However, there were no differences in the eccentricity value of CFA between both groups (0.74 ± 0.42 and 0.63 ± 0.28 mm; *P* = 0.195).

Moreover, Spearman correlation analysis showed that the square value of CFA was moderately negatively correlated with the type of OK lens (rs = − 0.366; *P* = 0.001) and moderately positively correlated with C12, including the change in front C12 (rs, 0.425; *p* < 0.001), change in total C12 (rs, 0.415; *p* < 0.001), front C12 (1-year; rs, 0.369; *p* = 0.001), and total C12 (1-year; rs, 0.401; *p* < 0.001).

### Factors associated with AL changes

The associations between the risk factors and changes in AL were analysed using bivariate logistic regression (Table 4). The covariates in the bivariate logistic regression model included the initial age at OK lens fitting, OK lens type, square value of CFA, total corneal surface of C8(1-year) and changes in the total corneal surface of C12. The results showed no significant change in AL between OK lens types (*P* = 0.079), square value of CFA (*P* = 0.56), total corneal surface of C8(1-year) (*P* = 0.546) and changes in the total corneal surface of C12 (*P* = 0.407). In contrast, the initial age at OK lens fitting significantly affected AL changes (*P* = 0.001).

**Table 4:** The associations between risk factors and changes in AL by bivariate logistics regression.

	B	S.E	Wald	Sig	Exp(B)	95% C.I.for EXP(B)	
						Lower	Upper
Age (years)	0.808	0.239	11.448	0.001	2.244	1.405	3.583
Type (CRT)							
Alpha	1.245	0.709	3.087	0.079	3.474	0.866	13.939
C8	− 1.71	2.834	0.364	0.546	0.181	0.001	46.736
C12	− 3.723	4.485	0.689	0.407	0.024	0	158.849
CFA	− 0.105	0.179	0.34	0.56	0.901	0.634	1.28
Constant	− 8.814	3.745	5.539	0.019	0		

C8: C8 on the total corneal surface (1-year). C12: the changes of C12 on the total corneal surface. *CFA* square value of central flattening area.

Age might be an independent factor affecting the ocular axis, but not the effect of OK lens. Therefore, the covariates in the second bivariate logistic regression model included only the OK lens type and changes in the total corneal surface of C12, as the initial age at OK lens fitting is not an effect of the OK lens, total corneal surface of C8 (1-year) (*P* > 0.5) and the square value of CFA was not significant (*P* > 0.5). Our results demonstrated a significant AL change between OK lens types (*P* = 0.031; Table 5).

**Table 5:** The associations between risk factors and changes in AL by bivariate logistics regression.

	B	S.E	Wald	Sig	Exp (B)	95% CI for Exp (B)	
						Lower	Upper
Type (CRT)							
Alpha	1.289	.599	4.635	.031	3.628	1.122	11.728
C12	− 5.583	3.628	2.368	.124	.004	.000	4.610
Constant	− 1.964	.797	6.073	.014	.140		

C12: the changes of C12 on the total corneal surface.

## Discussion

Our study found that the initial age of OK lens fitting, type of OK lens, SA, and central flattening area are factors affecting the myopia-controlling effect of OK lenses. In contrast to previous studies, we found that the type of OK lens and central flattening area might affect myopia control.

Our results indicate that the older a patient’s age at baseline, the smaller the axial elongation 1-year after wearing the OK lens (*P* < 0.001, Table 1). This finding is consistent with the findings of Wang^15^ and Santodomingo-Rubido^27^, who concluded that older myopic children at the initiation of OK lens wearing experienced slower AL growth than did younger children. However, even without OK lenses, the axial elongation rate decreases naturally with age^28^. Age may be the most important independent factor affecting axial growth. However, an OK lens can significantly delay axial progression^29^. Therefore, the effect of wearing OK lenses is predicted to be greater for older children with late-onset myopia.

Interestingly, OK lens type is the most important factor affecting myopia control if age as a factor is excluded. There are two main designs of OK lenses, namely VST and CRT. Brands of OK lenses include Menicon Z Night (Menicon Co., Ltd.), Alpha((Alpha Corp., Nagoya, Japan), Emerald (Euclid Systems Corporation, Herndon, VA), Euclid (Euclid Systems Corporation,USA), Hiline (Macro Vision, Taipei, Taiwan, China), and so on. We obtained CRT lenses from Paragon and VST lenses from Alpha, which displayed smaller axial elongation than the former lenses. Few studies have focused on this factor. Consistent with our results, Lu et al. found that VST lenses (brands: Euclid, Alpha, and Hiline) showed a better effect on myopia control but a weaker safety profile compared with the CRT lenses^30^. Moreover, Nakamura et al. found no differences among three types of VST lenses (brands: Menicon Z Night, Alpha, and Emerald)^31^.

The difference in the corneal shaping effect may be the main reason behind the difference in the myopia-controlling effect between the two OK lens designs. Our results show that the size of central the flattening area is moderately related with the type of OK lens and is significantly larger in the Paragon CRT lenses than in the Alpha OK lenses (VST design). Similarly, Marcotte-Collard found that the Paragon CRT lenses generated a greater diameter of the central flattening zone on the cornea than the Dream lenses (VST design), leading to possible differences in myopia management efficacy^32^. The central flattening area is surrounded by an annular high convex zone that introduces myopic defocus to control myopia progression. Our results and Marcotte-Collard’s findings demonstrate that the smaller the flattening area, the better the myopia control effect of the surrounding high convex zone.

Smaller CFA or the introduction of another myopic defocus of the CFA may be a step in the direction of improving OK lenses in the future. Paragon has launched a lens with a smaller optical zone (CRT 5.0 oz), which may have better myopia control. Multifocal OK, which has a dual-focus optics based on traditional OK lenses and, consequently, creates on-axis and peripheral myopic retinal defocus together, showed better myopia control than conventional OK lenses^33^. However, following the aforementioned changes, visual function declines at times. Nevertheless, ensuring both good distant vision and better myopia control effect is an aspect that needs further research.

In addition, high-order aberrations are another factor that affect myopia control. After OK treatment, the change in spherical aberrations (C12) becomes more negative, which may play a role in myopia progression. Our results showed that spherical aberrations were a relevant variable for axial elongation, consistent with the results of Lau^34^. The difference in the corneal shaping effect might be the main reason for the difference in spherical aberrations between both groups. Our results showed that the size of the central flattening area was moderately positively related with the change in spherical aberration (C12), and the size of the central flattening area was significantly smaller in the effective group than in the ineffective group. However, other studies reported different findings. For example, Hiraoka indicated that a coma aberration was a possible mechanism for slowing axial elongation in OK treatment^23^, but Santodomingo-Rubido showed that changes in corneal aberration were not significantly correlated with axial elongation^35^. Therefore, the influence of higher-order aberrations on ocular axis elongation requires further study.

A limitation of this study was its retrospective nature. The control and study groups were not randomised. Further randomised studies on the relationship between corneal shape and myopia-controlling effects among different designs of OK lenses are warranted as may help improve future OK lens designs.

In summary, the initial age at OK lens fitting, type of OK lens, size of CFA, and spherical aberration affect the myopia-controlling effect of OK lenses. Notably, different types of OK lenses have different effects on myopia control.

## Data Availability

The datasets used and/or analysed during the current study available from the corresponding author on reasonable request.

## References

[1] Grzybowski A, Kanclerz P, Tsubota K, Lanca C, Saw SM (2020). A review on the epidemiology of myopia in school children worldwide. BMC Ophthalmol..

[2] Jonas JB (2019). Myopia: Epidemiology, anatomy and prevention of myopia and treatment options for progressive myopia in childhood. Ophthalmologe.

[3] Recko M, Stahl ED (2015). Childhood myopia: Epidemiology, risk factors, and prevention. Mo. Med..

[4] SanzDiez P, Yang LH, Lu MX, Wahl S, Ohlendorf A (2019). Growth curves of myopia-related parameters to clinically monitor the refractive development in Chinese schoolchildren. Graefes Arch. Clin. Exp. Ophthalmol..

[5] Foo, L. L. *et al*. Predictors of myopic macular degeneration in a 12-year longitudinal study of Singapore adults with myopia. *Br. J. Ophthalmol*. (2022). 10.1136/bjophthalmol-2021-321046 35534177

[6] Tham YC (2016). Joint effects of intraocular pressure and myopia on risk of primary open-angle glaucoma: The Singapore epidemiology of eye diseases study. Sci. Rep..

[7] Dragoumis I, Richards A, Alexander P, Poulson A, Snead M (2017). Retinal detachment in severe myopia. Lancet.

[8] Haarman AEG (2020). The complications of myopia: A review and meta-analysis. Invest. Ophthalmol. Vis. Sci..

[9] Cho P, Cheung SW (2012). Retardation of myopia in Orthokeratology (ROMIO) study: A 2-year randomized clinical trial. Invest. Ophthalmol. Vis. Sci..

[10] Lee YC, Wang JH, Chiu CJ (2017). Effect of Orthokeratology on myopia progression: Twelve-year results of a retrospective cohort study. BMC Ophthalmol..

[11] Hiraoka T, Kakita T, Okamoto F, Takahashi H, Oshika T (2012). Long-term effect of overnight orthokeratology on axial length elongation in childhood myopia: A 5-year follow-up study. Invest. Ophthalmol. Vis. Sci..

[12] Faria-Ribeiro M, Belsue RN, Lopez-Gil N, Gonzalez-Meijome JM (2016). Morphology, topography, and optics of the orthokeratology cornea. J. Biomed. Opt..

[13] Zhong Y (2014). Corneal power change is predictive of myopia progression in orthokeratology. Optom. Vis. Sci..

[14] Chen C, Cheung SW, Cho P (2013). Myopia control using toric orthokeratology (TO-SEE study). Invest. Ophthalmol. Vis. Sci..

[15] Wang B, Naidu RK, Qu X (2017). Factors related to axial length elongation and myopia progression in orthokeratology practice. PLoS ONE.

[16] Yang X, Li Z, Zeng J (2016). A review of the potential factors influencing myopia progression in children using orthokeratology. Asia Pac. J. Ophthalmol. (Phila).

[17] Cho P, Cheung SW, Edwards M (2005). The longitudinal orthokeratology research in children (LORIC) in Hong Kong: A pilot study on refractive changes and myopic control. Curr. Eye Res..

[18] Kakita T, Hiraoka T, Oshika T (2011). Influence of overnight orthokeratology on axial elongation in childhood myopia. Invest. Ophthalmol. Vis. Sci..

[19] Chen Z (2012). Impact of pupil diameter on axial growth in orthokeratology. Optom. Vis. Sci..

[20] Joslin CE, Wu SM, McMahon TT, Shahidi M (2003). Higher-order wavefront aberrations in corneal refractive therapy. Optom. Vis. Sci..

[21] Berntsen DA, Barr JT, Mitchell GL (2005). The effect of overnight contact lens corneal reshaping on higher-order aberrations and best-corrected visual acuity. Optom. Vis. Sci..

[22] Downie LE, Lowe R (2013). Corneal reshaping influences myopic prescription stability (CRIMPS): An analysis of the effect of orthokeratology on childhood myopic refractive stability. Eye Contact Lens.

[23] Hiraoka T, Kakita T, Okamoto F, Oshika T (2015). Influence of ocular wavefront aberrations on axial length elongation in myopic children treated with overnight orthokeratology. Ophthalmology.

[24] Chen Z (2019). Adjunctive effect of orthokeratology and low dose atropine on axial elongation in fast-progressing myopic children-A preliminary retrospective study. Contact Lens Anterior Eye.

[25] Zhang Z (2022). Change in corneal power distribution in orthokeratology: A predictor for the change in axial length. Transl. Vis. Sci. Technol..

[26] Huang Z, Chen XF, He T, Tang Y, Du CX (2022). Synergistic effects of defocus-incorporated multiple segments and atropine in slowing the progression of myopia. Sci. Rep..

[27] Santodomingo-Rubido J, Villa-Collar C, Gilmartin B, Gutierrez-Ortega R (2013). Factors preventing myopia progression with orthokeratology correction. Optom. Vis. Sci..

[28] Hyman L (2005). Relationship of age, sex, and ethnicity with myopia progression and axial elongation in the correction of myopia evaluation trial. Arch. Ophthalmol..

[29] Walline JJ, Jones LA, Sinnott LT (2009). Corneal reshaping and myopia progression. Br. J. Ophthalmol..

[30] Lu W (2022). Comparison of two main orthokeratology lens designs in efficacy and safety for myopia control. Front. Med. (Lausanne).

[31] Nakamura Y (2021). Comparison of myopia progression between children wearing three types of orthokeratology lenses and children wearing single-vision spectacles. Jpn. J. Ophthalmol..

[32] Marcotte-Collard R, Simard P, Michaud L (2018). Analysis of two orthokeratology lens designs and comparison of their optical effects on the cornea. Eye Contact Lens.

[33] Loertscher M, Backhouse S, Phillips JR (2021). Multifocal orthokeratology versus conventional orthokeratology for myopia control: A paired-eye study. J. Clin. Med..

[34] Lau JK, Vincent SJ, Cheung SW, Cho P (2020). Higher-order aberrations and axial elongation in myopic children treated with orthokeratology. Invest. Ophthalmol. Vis. Sci..

[35] Santodomingo-Rubido J, Villa-Collar C, Gilmartin B, Gutierrez-Ortega R, Suzaki A (2017). Short- and long-term changes in corneal aberrations and axial length induced by orthokeratology in children are not correlated. Eye Contact Lens.

